# The effects of tv/video viewing hours on later ADHD symptoms: a counterfactual analysis in longitudinal population-representative data

**DOI:** 10.1186/s12887-025-05973-2

**Published:** 2025-09-01

**Authors:** Aja Murray, Hannah Casey, Helen Wright, Xinxin Zhu, Yi Yang, Xuefei Li, Zhuoni Xiao, Josiah King, Kasia Kostyrka-Allchorne, Edmund Sonuga-Barke

**Affiliations:** 1 Department of Psychology, University of Edinburgh, EH8 9JZ Edinburgh, UK; 2 Department of Psychiatry, University of Edinburgh, Edinburgh, UK; 3 Population Health Sciences Institute, Medical Sciences Faculty, Newcastle University, Edinburgh, UK; 4 Department of Psychology, School of Biological and Behavioural Sciences, Queen Mary University of London, London, UK; 5 Department of Child and Adolescent Psychiatry, Institute of Psychiatry, Psychology & Neuroscience, King’s College London, London, UK

**Keywords:** Screen time, Attention deficit hyperactivity disorder, TV/video viewing

## Abstract

**Background:**

It has been proposed that early-life screen use can impact the development of attention deficit hyperactivity disorder (ADHD) symptoms. Some studies have supported a weak association between higher levels of screen time and ADHD symptoms; however, this association is vulnerable to confounding and a causal explanation remains controversial.

**Methods:**

To help address confounding in this association, we conducted inverse probability of treatment weighting (IPTW) analyses in a large UK-representative longitudinal sample to examine the impact of TV/video viewing at age 3 on ADHD symptoms at age 5.

**Results:**

Adjusting for confounding, we found that both no TV/video viewing and viewing more than 3 h of TV/video compared to between 1 and 3 h were associated with increased ADHD symptoms. However, the effect of no TV/video viewing was mirrored in a negative outcome control analysis, suggesting potential residual confounding.

**Conclusions:**

Results are consistent with claims that high levels of TV/video viewing in preschool years may impact the development of ADHD symptoms. Trial-based research which examines the impact of reducing high levels of TV/video viewing in this age group would be merited to further illuminate this association and assess whether it is likely to reflect a causal effect.

**Clinical trial number:**

Not applicable.

**Supplementary Information:**

The online version contains supplementary material available at 10.1186/s12887-025-05973-2.

Attention deficit hyperactivity disorder (ADHD) symptoms relate to two broad domains: inattention (e.g., difficulties in concentration) and hyperactivity/impulsivity (e.g., physical restlessness, acting before thinking) [1]. It has been proposed that screen use, such as television (TV)/video viewing, can impact the development of ADHD symptoms, with varied mechanisms claimed to contribute to the link [2].

Despite a range of plausible mechanistic hypotheses, empirical investigations have fallen short of definitively demonstrating a causal link between screen use and ADHD symptoms. Though a majority of studies support a link (of modest effect size), evidence for an association is nevertheless somewhat mixed [3–10]. Most studies have also relied on cross-sectional designs, making it difficult to ascertain direction(s) of causality [5, 11]. A recent review of 28 longitudinal studies concluded that, overall, there was evidence for reciprocal relations between digital media use (defined for the purposes of the review as gaming and social media and including both screen time and problematic use) and ADHD. However, the relation between screen time (i.e., time spent using one or more types of digital media) specifically and ADHD symptoms was not universally replicated [11]. A recent systematic review examining the links between screen time in preschoolers and later attention problems found that a majority of, but not all, longitudinal studies supported an association [12]. However, significant associations between screen use and ADHD symptoms have been seen to be attenuated to statistical non-significance when adjusting for baseline measures [11], or confounders [5, 13]. The effect of TV/video screen time as a specific form of screen use remains similarly unclear.

A critical issue in understanding the potential effects of TV/video viewing on ADHD symptoms is that the association may be confounded by factors that impact both, such as sex/gender, family resources, parenting, and child temperament, especially early temperaments that could represent the precursors to ADHD symptoms [11]. Randomised controlled trials (RCTs) that could help address confounding are lacking in this field and it has been noted that they are challenging to conduct [11]. However, counterfactual analysis approaches in longitudinal data can help address confounding by providing a framework for conceptualising potential confounding variables and accounting for them in empirical analyses [14, 15].

Where measures of confounding variables (or good proxies) are available, propensity score based counterfactual analyses provide a set of tools for accounting for their effects [16]. Specifically, measured confounders can be used to estimate propensity scores for individuals in the sample, reflecting the extent to which they were likely to have received the ‘exposure’ or ‘treatment’ (e.g., TV/video viewing). These scores can then provide the basis for matching, stratifying, or weighting individuals to ‘re-balance’ exposed and non-exposed groups (or groups exposed to different levels of an exposure variable) with respect to the confounders to facilitate a comparison of their outcome scores (e.g., ADHD symptoms). When the assumptions of the respective approaches are met, RCTs achieve balance on confounders through randomisation, whereas propensity score approaches achieve it through the matching, weighting or otherwise ‘re-balancing’ of the confounders across groups. Utilising counterfactual analysis approaches can thus help address recent calls for studies that investigate causality in the effects of screen use on ADHD symptoms [2].

The use of negative controls within a counterfactual analysis approach provides a further means to gain insights into associations that are vulnerable to the effects of confounding and other biases in observational studies [17, 18]. In essence, negative controls are used to recreate conditions that do not involve the hypothesised causal mechanism (here: the effects of TV/video viewing on ADHD symptoms) but are likely to involve the same biases (e.g., the same confounders as might be present in the TV/video viewing and ADHD symptoms associations). Negative *outcome* controls are variables that are not thought to be causally impacted by the exposure of interest while following a shared confounding mechanism with the exposure and outcome variables of interest. For example, a child’s video/TV viewing time would not be expected to strongly influence their levels of prosocial behaviour later [19] but this association may be affected by similar biases (shared confounding variables, socially desirable responding by parents) and so could be used as a negative outcome control. If the negative outcome control variable shows an association with the exposure (TV/video viewing) after adjusting for confounding, this implies that the primary association of interest (that between TV/video viewing and ADHD symptoms) may be affected by residual confounding and/or other biases in common with the primary outcome. Comparing the primary and negative control associations can, therefore, provide insights into the nature and extent of biases in the primary outcome analysis. Previous examples of negative control use in ADHD research include using acetaminophen before and after pregnancy as negative controls when examining the effects of its use in pregnancy on child ADHD [20] and using paternal substance use as well as maternal substance use outside of pregnancy as negative controls when examining the effects of maternal substance use in pregnancy on child ADHD [21]. However, the use of negative controls in psychological research overall remains uncommon.

## The present study

In this study we apply a counterfactual analysis approach to address confounding and explore the association between TV/video viewing and later ADHD symptoms in a large UK representative study. We focus on preschool-age screen time because it has been proposed that this may represent an especially sensitive period of development for the effects of screen use due to greater brain plasticity [8, 11]. We focus on TV/video use as the predominant type of screen use in this age group [22–24] and include a negative outcome control to provide further insights into the extent to which the exposure effects suggest strong evidence for an effect of video/TV viewing in toddlerhood on the development of ADHD symptoms.

Method.

## Method

### Participants

Participants were drawn from the Millennium Cohort Study (MCS) [25]. MCS is a longitudinal cohort of children born in the United Kingdom (UK) at the beginning of the 21 st Century. At baseline, participants were sampled from the nations of the UK using stratified random sampling. Families living in areas of high ethnic minority concentration and of greater social disadvantage were oversampled. Design weights are then provided to adjust for the complex sampling design in order that the resulting (weighted) estimates can be treated as population representative.

The data and associated documentation are available via the UK data service: https://ukdataservice.ac.uk. Data from baseline (wave 1, collected in 2001) up to age 5 (wave 3, collected in 2006) were used in the current analysis. The exposure variable (TV/video viewing) was taken from wave 2 (collected in 2004), matching variables (hypothesised confounders) were taken from wave 1, and the outcome variable (ADHD symptoms) was from wave 3.

At baseline, the sample included *n* = 9634 boys and *n* = 9151 girls. The majority of the sample’s ethnic group was classified based on UK census categories as White (*n* = 15,491), with *n* = 526 classified as Mixed; *n* = 470 as Indian; *n* = 1271 as Pakistani and Bangladeshi; *n* = 678 as Black or Black British; and *n* = 266 as Other Ethnic Group. In terms of socioeconomic status, the breakdown of parental highest academic qualification were: *n* = 627 for higher degree; *n* = 2318 for first degree; *n* = 1585 for diploma in higher education; *n* = 1737 for A/AS/S levels; *n* = 6260 for O level/GCSE grades A-C; *n* = 1998 for GCSE grades D-G; *n* = 535 for other academic qualifications and *n* = 3655 for none of these qualifications, while the distribution of family income was as follows: *n* = 301 in the <£3100 per annum bracket; *n* = 4457 in the £3100 - <£10,400 bracket; *n* = 5707 in the £10, 400 to < £20,800 bracket; *n* = 3364 in the £20,800 to < £31,200 bracket; *n* = 2445 in the £31,200 to < £52,000 bracket; *n* = 879 in the £52,000 and above bracket. Specific effective sample sizes are provided below for each analysis.

MCS obtained approval from the UK National Health Service Research Ethics Committee, and participating parents/young people (as relevant) provided written consent at each survey.

### Measures

*TV/video viewing* at age 3 was the exposure variable in this analysis and was measured based on a single self-report item included as part of parent/caregiver interviews about their child. Parents/caregivers were asked to report the number of hours their child spent TV/video viewing per day, using the response options: *not at all; up to one hour; more than one hour*,* less than three hours; more than three hours.* A *not applicable* option was also available.

*ADHD symptoms* at age 5 were the outcome variable and were measured using parent-reported hyperactivity/inattention subscale from the *Strengths and Difficulties Questionnaire (SDQ*) [26]. The SDQ is a widely used and well-validated instrument for use in children [27]. In the current sample it has shown good psychometric properties, including a high degree of gender, informant and developmental invariance [28, 29]. The hyperactivity/inattention subscale has also shown good discrimination with respect to ADHD diagnosis [30, 31], despite some debate regarding the most appropriate cut-off point to indicate clinically significant symptoms. The subscale includes five items that refer to behaviour during the last six months with reference to the following behaviours: ‘restless, overactive, cannot stay still for long’; ‘constantly fidgeting or squirming’; ‘easily distracted, concentration wanders’; ‘thinks things out before acting’; and ‘sees tasks through to the end, good attention span’. Responses were provided on a 3-point Likert-type scale with options: *not true* (0), *somewhat true* [1], and *certainly true* [2]. A *can’t say* response option is also offered. Positively worded items were reverse-coded, and item responses were summed to produce an overall hyperactive/inattentive score with higher scores indicating greater hyperactivity/inattention (possible range = 0–10).

Fuller details of matching variables are provided in Supplementary Materials. These were *child sex*,* parental education* (highest academic qualification), *prematurity* (gestational age at birth less than 259 days), *low birth weight* (birth weight less than 2.5 kg), *poverty indicator*,* temperament* (mood, adaptability, regularity and fuss), *early parenting* (views on importance of regular patterns, cognitive stimulation, and talking to child) and *parental mental health*.

*Prosociality* at age 5 was included as a negative outcome control and was measured using the parent-reported prosociality subscale of the SDQ. Its items are “considerate of other people’s feelings” “shares readily with other children (treats, toys, pencils etc.)”, “is helpful if someone is hurt, upset or feeling ill”, “is kind to younger children”, and “often volunteers to help others (parents, teachers, other children)”. The response scale is as described for the hyperactivity/inattention subscale above.

### Variable selection

#### Matching variable selection

Matching variables for the propensity score model were selected based on principles articulated by VanderWeele [32], namely, variables should be causes of both the exposure (TV/video viewing hours) and outcome (ADHD symptoms) or a proxy for an unmeasured common cause. This implied that all matching variables occurred prior to the exposure (in practice meaning that they were taken from the baseline wave of MCS). Instrumental variables, i.e., variables that are causes of the exposure and only related to the outcome via their relation to the exposure, were excluded from the set of matching variables as their inclusion can amplify bias in the presence of residual confounding.

#### Negative outcome control

Based on data availability, a negative control was additionally selected and utilised in additional analyses. Prosociality was selected as a negative outcome control as: (i) its association with TV/video viewing is likely to be affected by the same third variables (ii) evidence suggests that whilst certain formats and contents may promote prosociality in toddlers, video/TV screen time in itself is not likely to be an active ingredient [19] (iii) prosociality is measured by the same survey instrument and by the same informant as ADHD symptoms, namely the parent-reported *Strengths and Difficulties Questionnaire (SDQ)*, meaning that its inclusion can additionally help gauge the effect of bias due to rater effects. Given this, any confounder-adjusted association between video/TV viewing and prosociality would be expected to be null or at least considerably smaller than with ADHD. If not, this would indicate potential remaining bias in the ADHD analyses. A suitable negative exposure control (i.e., an exposure sharing the confounding structure of the primary analyses but assumed not to be causally related to ADHD symptoms) could not be identified in the dataset.

### Statistical analysis

Analyses were based on the DigiCAT workflow [33], which brings together and augments R packages for counterfactual analysis and aims to embed best practices in propensity estimation, balance checking, missing data treatment, incorporation of complex survey design variables, outcome model fitting, and marginal effects estimation when treatment by matching variable interactions are included in the outcome model. Effect sizes (Hedges’ *g* [34]) were calculated for each contrast, incorporating weighting where relevant. These can be interpreted using the conventions of small effect size = 0.2, medium = 0.5, and large = 0.8. R code is provided at: OSF | TV_video impact OSF.R.

#### ADHD and TV/video viewing analyses

We began by fitting design-adjusted linear regression models without adjustment for any covariates, to estimate the ‘uncorrected’ association between hours watching TV/video at age 3 and hyperactivity/inattention at age 5. We present the results in terms of the comparison of the effects of different levels of TV/video watching to the category, i.e. *more than one hour*,* less than three*, to provide the greatest interpretability of findings (see Results). This was included because, by comparison with the IPTW analyses, it can provide a sense of the extent to which unadjusted analyses of the effects of TV/video viewing on ADHD symptoms may be biased.

As our main analyses, we used an inverse probability of treatment weighting (IPTW) approach to account for confounding, using the WeightIt package in R [32]. Given the ordinal measurement scale of the treatment variable, propensity scores were estimated using ordinal logistic regression. Average treatment effect (ATE) weights were derived from these propensity scores and multiplied by the attrition weights for application in the outcome model. Covariate balance was checked by examining the maximum difference in matching variables across exposure-level pairs. For continuous matching variables, the standardised mean difference across exposure level pairs was used and for binary and nominal variables, the raw difference in proportion was used.

If matching was successful, a weighted linear regression model was fit, in which the outcome variable (ADHD symptoms) was regressed on the exposure variable (TV/video viewing), the matching variables, and the interactions between the matching variables and the exposure. The effect of TV/video viewing was then estimated from this model using a marginal effects calculation to compare different levels of the exposure variable on ADHD symptoms. For comparison, a model with attrition adjustment only and with attrition and IPTW adjustment (but no additional covariates in the outcome model) was also fitted. This allowed us to assess the impact of increasingly stringent confounding control on the association between TV/video viewing and ADHD symptoms.

An analysis of linear, quadratic and cubic trends was conducted using ordinal contrasts to gain further insights into potential non-linear associations, except in the fully adjusted model with interactions due to the complexity of interpreting the trends in the context of interactions. These analyses provide complementary information that aids in the interpretation of the main analysis.

#### Negative control analyses

A negative outcome control model was fit in which prosociality was regressed on TV/video viewing in an IPTW-weighted regression. In this model, matching variables and their interactions with the treatment variable were included as covariates. Marginal effects were calculated to provide the effect associated with TV/video viewing.

## Results

### Descriptive statistics

The mean (design- and attrition-weighted) ADHD symptom score at age 5 in the sample was 3.28 (SD = 2.37), while the mean (design- and attrition-weighted) prosociality score at the same age was 8.38 (SD = 1.67). The weighted frequencies of watching TV/videos at age 3 were: *not at all*: *n* = 147; *up to one hour*: *n* = 3058; *more than one hour*,* less than three hours*: *n* = 8200; *more than three hours*: *n* = 2334.

### Attrition-adjusted regressions

Analyses adjusted only for the survey design variables and no confounders are provided in Table 1. These suggested that compared to watching between one and three hours, watching *none at all* and *more than three hours* was associated with increased ADHD symptoms, whilst watching *up to one hour* was associated with decreased ADHD symptoms. Consistent with this, an analysis of the linear, quadratic and cubic trends associated with increasing TV/video watching time suggested no significant linear effect (B = −0.13, *p* =.46) but a significant quadratic (B = 0.83, *p* <.001) and cubic (B= −0.20, *p* =.005) effect. Overall, these results suggest that optimal TV/video viewing time with respect to ADHD symptoms for this age group is up to one hour.

**Table 1:** Association between tv/video viewing at age 3 and ADHD symptoms at age 5

	Attrition-adjusted					IPTW and attrition-adjusted					Main analysis: IPTW and attrition-adjusted with treatment by matching variable interactions*				
	B	SE	t	*p*	g	B	SE	t	*p*	g	Marginal effect	SE	z	*p*	g
Intercept	3.178	0.034	92.959		-	3.160	0.035	91.323		-	-	-	-	-	
None at all	0.868	0.267	3.248	0.001*	0.376	1.512	0.317	4.768		0.653	1.080	0.332	−3.260	0.001*	0.466
Up to one hour	−0.203	0.065	−3.130	0.002*	−0.088	0.108	0.072	1.492	0.137	0.046	−0.020	0.087	−0.230	0.818	−0.009
More than three hours	0.604	0.066	9.194		0.259	0.440	0.067	6.613		0.190	0.350	0.095	3.690		0.151

More than one hour, less than three hours is the reference category for TV/video viewing at age 3, *SE* standard error, *When estimating the model including matching variables and treatment by matching variable interactions, it was necessary to remove the interactions between the treatment and highest educational level due to model estimation issues, *g* Hedge’s *g* effect size, * significant at *p* <.05

### IPTW- and attrition-adjusted regressions

Matching statistics (the maximum difference in SMD or proportion across treatment variable levels) for each matching variable after IPTW-weighting are provided in Table S2. The maximum difference in proportions was for the *first degree* level of academic qualifications (difference in proportion = 0.337) but the other levels of this variable were also not well balanced (smallest maximum proportion difference for this variable was 0.21). All other variables appeared to be well-balanced between different levels with maximum proportion differences < 0.12 and SMDs < 0.05. The effective sample sizes after weighting were: *not at all*: *n* = 131; *up to one hour*: *n* = 2519; *more than one hour*,* less than three hours* *n* = 7795; and *more than three hours* *n* = 2034.

Analyses adjusted for both the survey design variables and confounders using IPTW but without additional adjustment for the matching variables in the outcome model are provided in Table 1. These suggested that compared to watching *more than one hour*,* less than three hours* of video/TV daily, watching *none at all* and watching *more than three hours* were associated with increased ADHD symptoms. Watching *up to one hour per day* was not significantly different in terms of later ADHD symptoms to watching *between one and three hours*. An analysis of the linear, quadratic and cubic trends suggested a significant linear (B= −74, *p *001), quadratic (B = 0.92, *p* <.001) and cubic trend (B = −0.17, *p* =.047). Overall, these analyses suggested that no TV/video at all or more than three hours of daily TV/video were associated with increased ADHD symptoms relative to watching between one and three hours.

In the main analysis, when including matching variables and their interactions with the exposure as additional covariates in the model, compared to watching between one and three hours of TV/video, watching *none at all* and watching *more than three hours* were associated with increased ADHD symptoms. The effects were attenuated relative to the model with IPTW and attrition adjustment only, suggesting that more stringent control was necessary for this association. There was no significant effect of watching *up to an hour* compared to watching between one and three hours.

### Negative control analyses

Results of the negative outcome control analyses are provided in Table 2 and suggested that watching no TV at all (*none at all*) compared to watching between one and three hours was associated with decreased prosociality. There were no other significant effects. The effect of watching no TV showed a similar pattern to the ADHD analyses and may be indicative of residual bias in the latter. However, these analyses did not replicate the negative effects of watching *more than three hours* of TV on ADHD symptoms.

**Table 2:** IPTW-attrition adjusted negative outcome control (TV/video effects on prosociality)

	B	SE	*P*	g
None at all	−0.877	0.251		−0.524
Up to one hour	0.037	0.057	0.520	0.022
More than three hours	0.091	0.065	0.163	0.054

More than one hour, less than three hours is the reference category for TV/video viewing at age 3, *SE* standard error, *When estimating the model including matching variables and treatment by matching variable interactions, it was necessary to remove the interactions between the treatment and highest educational level due to model estimation issues, *g* Hedge’s *g,* * significant at *p* <.0.5

## Discussion

Given concerns about the potential impact of early screen time on the development of ADHD symptoms coupled with the difficulty of estimating these effects in the context of confounding challenges, we applied a counterfactual analysis approach to examine the effects of TV/video viewing on the development of ADHD symptoms ~ 2 years later in a large UK-based longitudinal study. We focused on the effects of TV/video viewing at age 3 on ADHD symptoms at age 5, given that this has been previously argued to represent a sensitive period of development with respect to screen time effects [8]. Results suggested that the effects are not straightforward: compared with watching between 1 and 3 daily hours of TV/video, watching *more than 3 hours* and watching *none at all* were both associated with increased ADHD symptoms. Additional negative outcome control analyses reproduced the effect of not watching TV/video at all, suggesting potential residual bias in this effect and thereby calling the result into question. However, they did not reproduce the negative effect of watching more than 3 hours of TV/video.

Very few previous studies have used methods tailored for causal inference to investigate the links between TV/video viewing and ADHD symptoms. However, our findings are consistent with one previous study that found some evidence of a causal link between TV/video viewing and ADHD using a Mendelian randomisation approach [35]. They reported an increased risk of childhood ADHD associated with time spent watching TV (OR = 2.1) but no evidence for an effect of childhood ADHD in a reverse analysis. This is consistent with a causal impact of TV viewing on ADHD but not an effect in the opposite direction. Our results also reveal subtleties to the biases that may be present when potential confounding factors are not adjusted for. Specifically, we found that when only adjusting for attrition but not confounding, all comparisons with ‘moderate’ levels of screen use (up to one hour) were significant. With adjustment for confounding, the effect in the comparison with no screen use becomes stronger; however, the comparison with high levels of screen time becomes attenuated. The latter effect suggests potential interactions in the patterns of confounding present for this association.

Our results are also largely consistent with previous meta-analytic studies that have suggested small positive correlations between media use and ADHD symptoms [36]. Previous longitudinal studies that have addressed confounding through more traditional methods such as regression adjustment have also shown a positive relation between screen use and later ADHD symptoms [11]. However, IPTW has some advantages over these methods in that it employs an explicitly counterfactual framework that guides the identification and inclusion of pre-exposure confounding factors, includes balance checking steps to ensure that the exposure groups are well-balanced (achieved through weighting), and can be combined with regression adjustment for matching variables and their interaction with the exposure group in the outcome model to provide ‘double robustness’. Our negative control analyses also helped evaluate whether there was likely to be residual confounding in the association.

A relatively more novel nuance also emerged, namely that moderate amounts of TV/video viewing (1–3 h) were associated with *lower* levels of ADHD symptoms than with both large amounts (3 + hours) and with none at all. One possibility is that the finding of increased ADHD symptoms associated with no TV/video viewing reflects some residual confounding. For example, overly active toddlers may not be attracted to TV/video viewing even if caregivers offer them the opportunity to engage in TV/video viewing. Alternatively, children who do not watch any TV/video may have pre-existing health or neurodevelopmental concerns that could also impact later ADHD symptoms. The hypothesis that this association reflects residual confounding is consistent with our negative outcome analyses which replicated this effect for an outcome that would not be expected to be associated with TV/video viewing time based on past research [19]. However, there are other possibilities. For example, some theories of ADHD propose that the condition is characterised by relative hypoarousal and various models build on this to suggest that external stimulation can improve symptoms [37, 38]. If TV/video provides an arousal-regulating source of external stimulation, children at risk of ADHD could potentially benefit from some TV/video viewing relative to none. However, further research will be required to better illuminate the underlying reasons for this finding. On a methodological note, the finding also underlines the importance of allowing for the possibility of non-linear effects of screen use [13].

The large, representative nature of the MCS sample is advantageous with respect to estimating screen time effects on ADHD symptoms for UK children and the estimates from this sample suggested that magnitude of the effects may be quite modest. Specifically, the effect associated with watching > 3 h of TV/video daily compared with between 1 and 3 was 0.350, where the possible score range is 0 to 10. In terms of effect size, this translated into a small effect size. However, the presence of negative effects is in line with existing guidelines that recommend TV/video viewing limits for young children. Specific guidelines vary but, for example, the World Health Organization (WHO) recommends that children aged 3–4 years limit sedentary screen time to no more than 60 min per day (World Health Organization, 2019). As screen use remains high among this age group despite these guidelines (with the largest proportion of the current sample watching between 1 and 3 h per day), further research may be needed to help inform effective public health strategies to help parents/caregivers to sustainably reduce their children’s TV/video viewing.

### Future directions

There are several future research directions that can build on the present findings. First, despite the challenges highlighted in conducting interventions in reducing screen time, it will be important to establish the extent to which trial-based research on preschool TV/video viewing aligns with counterfactual analyses from observational data. Trial and observational counterfactual approaches have complementary strengths and typically exhibit different types of biases [39], therefore, triangulation across these methods, including when observational studies are explicitly designed to emulate a target trial can help bolster claims that the effects are causal [40].

Second, to the extent that the effect is causal, further research is needed to illuminate the active ingredients in TV/video viewing. Several different explanations have been proposed; however, differentiating them requires accurate measurement of aspects of TV/video viewing such as passive vs. active viewing, violent versus non-violent (or otherwise ‘active’/impulsive) content, and pacing [2]. However, whilst lab-based studies may be capable of isolating these aspects, this is at the expense of ecological validity and it is unclear how effects identified in such settings generalise to the cumulative effects of viewing in the course of real life. Innovations in capturing ‘real-life’ TV viewing and affective, cognitive, and behavioural reactions in ecological context over extended periods of time may be needed to substantively advance understanding of these issues. Candidate mediators such as sleep and neuropsychological changes such as delay aversion, sleep displacement would also be valuable to capture.

Third, it will be valuable to explore the mechanisms linking screen use to ADHD symptoms. For example, the ‘scan and shift’ hypothesis suggests that the fast pace of digital media promotes an attentional style characterised by rapid shifts of attention, undermining the ability to deploy sustained attention when required [36]. Another perspective posits that high levels of screen time might undermine children’s ability to internally regulate their attention due to becoming accustomed to external regulation via media content [41]. A further proposal is that high arousal resulting from screen use may result in habituation and subsequent difficulties in engaging in low arousal tasks [11]. Relatedly, it has been proposed that viewing violent content may result in children displaying impulsive and hyperactive behaviours via observational learning [36]. Finally, rather than fundamentally altering brain development, it has been suggested that digital exposure may create ‘phenocopies’ of ADHD by favouring and reinforcing ADHD-like patterns of attending in neurotypical young people [42].

Fourth, it has been proposed that the effects of screens may differ based on individual vulnerabilities [2], therefore, future research would be valuable to explore moderators of the effects identified in the present study. Research points to sex/gender, parenting, and parental mental health as potential moderators [2]; however, others could potentially be identified using exploratory approaches such as causal trees or forests [43]. Importantly, it has been hypothesised that ADHD symptoms themselves may increase susceptibility to the effects of screens [8].

Finally, while the scope of the present study concerned the effects of early TV/video viewing on later ADHD symptoms, a developmental perspective focused on illuminating their linkages over time should attend to potential reciprocal effects. This is in line with the hypothesis that individuals select into viewing habits that are consistent with and which may serve to reinforce pre-existing dispositions. For example, it has been hypothesised that those with higher levels of ADHD symptoms may have a preference for faster paced/higher action TV/video viewing, which is one factor which has been proposed to be responsible for the effects of TV/video viewing on ADHD symptoms.

### Limitations

It is important to note the limitations of the present study. First, despite the availability of a wide range of matching variables in this study, there is always a possibility of unmeasured confounding in counterfactual analysis of observational data leading to violation of the ‘unconfoundedness’ assumption of the IPTW methods we used. A primary limitation in this respect is that while we included child temperament to capture features that may represent precursors to ADHD symptoms, we did not include pre-exposure ADHD symptoms themselves in our set of matching variables because they are difficult to measure at very young ages. There are also other assumptions underlying IPTW that could not be directly tested. This includes the ‘stable unit treatment value assignment (SUVTA)’ assumption comprising the assumption that there is only a single version of the treatment/exposure and that treatment assignment for units do not affect the treatment assignment of others. It also includes the positivity assumption (that each unit has a positive probability of receiving the treatment/exposure) and consistency (that the potential outcome under a particular treatment/exposure is equal to the observed outcome if that treatment/exposure is actually received). Finally, though we used a negative outcome control to help provide further information on potential biases in the primary association, we used a somewhat ambiguous control in the sense that the true causal relation between TV/video effects and prosociality is not in itself fully understood. This complicates the interpretation of the comparison between the primary and negative outcome control analysis and permits us to use it as further ancillary triangulating evidence rather than a definitive control.

Another important limitation was the TV/video viewing measure, which focused on screen time and used a single item. We had no information about the content of TV/videos viewed, which as noted above may be important in its effects [44]. Further, we relied solely on parental reports, which had three primary disadvantages: parents may not accurately recall TV/video hours watched, there may be inflated associations between the exposure and outcome due to ‘common rater effects’ [45]; and the lack of multi-informant perspective that is the gold standard in the assessment of child neurodevelopmental and mental health outcomes [46]. For example, previous research has shown how parents and teachers may disagree on ADHD symptoms, yet each perspective can provide unique valid information (e.g., about behaviour in different settings) [29, 47, 48]. Finally, the reference period was undefined for the TV/video viewing was =, creating further potential for ambiguity and individual differences in interpretations of the item.

## Conclusions

Our results provide support from a counterfactual analysis approach that high levels of TV/video viewing in the preschool years are related to the later ADHD symptoms. No TV/video viewing compared to low levels was also associated with increased ADHD symptoms; however, negative control analyses suggested that this might be due to the presence of residual confounding. Future research could adopt a similar paradigm to illuminate the effects of TV/video viewing going beyond mere screen time. The results suggest that further intervention-based research would be merited to help establish whether the findings reported here reflect causality.

## Supplementary Information

Supplementary Material 1.

## Data Availability

The datasets used and/or analysed during the current study are available via the UK data service: https://ukdataservice.ac.uk.

## Abbreviations

ADHD: Attention Deficit Hyperactivity Disorder
MCS: Millennium Cohort Study
IPTW: Inverse Probability of Treatment Weighting
SDQ: Strengths and Difficulties Questionnaire
ATE: Average Treatment Effect
