# Supplementary material for: The effects of tv/video viewing hours on later ADHD symptoms: a counterfactual analysis in longitudinal population-representative data
Source: BMC Pediatr. 2025 Sep 1;25:673. doi: 10.1186/s12887-025-05973-2 (PMC12400586; doi:10.1186/s12887-025-05973-2)
Supplement: Supplementary file 1 — Supplementary Material 1. [file 12887_2025_5973_MOESM1_ESM.docx]

**Supplementary Materials**

**Table S1: Variables included in analysis**

| **MCS Dataset** | **MCS variable label** | **MCS variable description** | **Variable derivations** |
| --- | --- | --- | --- |
| mcs1_parent_interview.sav | APACQU00 | Highest academic qualification |  |
| mcs1_cm_derived.sav | ADBWGT00 | DV Cohort Member birth weight in kilos | Used to derive low birth weight (LBW) |
| mcs1_cm_derived.sav | ADGEST00 | DV Cohort Member Gestation time in days | Used to derive Prematurity (prem) |
| mcs1_family_derived.sav | ADOEDP00 | DV OECD below 60% median poverty indicator |  |
| mcs1_parent_cm_interview.sav | ACBFEV00 | Ever tried to breastfeed |  |
| mcs1_parent_cm_interview.sav | APHAPN00 | Happy sounds during nappy changing etc | Used to derive mood composite  (temp_mood) |
| mcs1_parent_cm_interview.sav | APUNFA00 | Pleasant first arriving in unfamiliar places. |  |
| mcs1_parent_cm_interview.sav | APBRUS00 | Pleasant during hair brushing etc |  |
| mcs1_parent_cm_interview.sav | APFEED00 | Content during interruptions of feeding. |  |
| mcs1_parent_cm_interview.sav | APINJU00 | Pleasant or calm with minor injuries |  |
| mcs1_parent_cm_interview.sav | APBATH00 | Objects to bathing - different place/person | Used to derive adaptability composite (temp_adapt) |
| mcs1_parent_cm_interview.sav | APWARY00 | Wary of strangers after 15 minutes. |  |
| mcs1_parent_cm_interview.sav | APBSHY00 | Shy on first meeting another child |  |
| mcs1_parent_cm_interview.sav | APFRET00 | Fretful in a new place or situation |  |
| mcs1_parent_cm_interview.sav | APSLEE00 | Bothered at first by different sleeping place. |  |
| mcs1_parent_cm_interview.sav | APMILK00 | Milk feeds at about the same time | Used to derive regularity composite (temp_reg) |
| mcs1_parent_cm_interview.sav | APSLTI00 | Sleepy at about the same time each evening |  |
| mcs1_parent_cm_interview.sav | APNAPS00 | Naps about the same length |  |
| mcs1_parent_cm_interview.sav | APSOFO00 | Solid food at about the same time |  |
| mcs1_parent_cm_interview.sav | APFUBS00 | Makes a fuss or cry before going to sleep | Used to derive fuss composite (temp_cry) |
| mcs1_parent_cm_interview.sav | APFUAS00 | Make a fuss or cry shortly after waking up |  |
| mcs1_parent_cm_interview.sav | APCRYS00 | Becomes upset when does not get what wants |  |
| mcs1_parent_interview.sav | APPRPL00 | Planned pregnancy |  |
| mcs1_parent_interview.sav | APILPR00 | Any illnesses or problems during pregnancy |  |
| mcs1_parent_interview.sav | APDEAN00 | Whether ever diagnosed with depresion/serious anxiety |  |
| mcs1_parent_interview.sav | APFPAT00 | Regular sleeping and eating |  |
| mcs1_parent_interview.sav | APSTIM00 | Stimulation for development |  |
| mcs1_parent_interview.sav | APTALK00 | Importance of talking |  |
| mcs1_parent_interview.sav | APTIRE00 | Tired most of time |  |
| mcs1_parent_interview.sav | APDEPR00 | Often miserable or depressed | Used to derive parental mental health (parentalMH) |
| mcs1_parent_interview.sav | APWORR00 | Often worried about things |  |
| mcs1_parent_interview.sav | APRAGE00 | Often gets in violent rage |  |
| mcs1_parent_interview.sav | APSCAR00 | Suddenly scared for no good reason |  |
| mcs1_parent_interview.sav | APUPSE00 | Easily upset or irritated |  |
| mcs1_parent_interview.sav | APKEYD00 | Constantly keyed up or jittery |  |
| mcs1_parent_interview.sav | APNERV00 | Every little thing gets on nerves |  |
| mcs1_parent_interview.sav | APHERA00 | Heart often races like mad |  |

**Table S2: Balance statistics for TV/video viewing**

| **Matching variable** | **Variable scale** | **Adjusted SMD /difference in proportion** |
| --- | --- | --- |
| **AHCSEX00_2** | Binary | 0.026 |
| **APACQU00_1** | Binary | 0.010 |
| **APACQU00_2** | Binary | 0.091 |
| **APACQU00_3** | Binary | 0.058 |
| **APACQU00_4** | Binary | 0.024 |
| **APACQU00_5** | Binary | 0.048 |
| **APACQU00_6** | Binary | 0.048 |
| **APACQU00_95** | Binary | 0.029 |
| **APACQU00_96** | Binary | 0.216 |
| **APACQU00:<NA>** | Binary | 0.003 |
| **prem** | Binary | 0.008 |
| **prem:<NA>** | Binary | 0.008 |
| **LBW** | Binary | 0.038 |
| **LBW:<NA>** | Binary | 0.002 |
| **ADOEDP00** | Binary | 0.337 |
| **ADOEDP00:<NA>** | Binary | 0.003 |
| **ACBFEV00_2** | Binary | 0.123 |
| **ACBFEV00:<NA>** | Binary | 0.001 |
| **temp_mood** | Continuous | 0.134 |
| **temp_mood:<NA>** | Binary | 0.035 |
| **temp_adapt** | Continuous | 0.314 |
| **temp_adapt:<NA>** | Binary | 0.057 |
| **temp_reg** | Continuous | 0.295 |
| **temp_reg:<NA>** | Binary | 0.022 |
| **temp_cry** | Continuous | 0.033 |
| **temp_cry:<NA>** | Binary | 0.020 |
| **APPRPL00_2** | Binary | 0.086 |
| **APPRPL00:<NA>** | Binary | 0.002 |
| **APILPR00_2** | Binary | 0.025 |
| **APILPR00:<NA>** | Binary | 0.002 |
| **APDEAN00_2** | Binary | 0.048 |
| **APDEAN00:<NA>** | Binary | 0.000 |
| **APFPAT00** | Continuous | 0.268 |
| **APFPAT00:<NA>** | Binary | 0.012 |
| **APSTIM00** | Continuous | 0.251 |
| **APSTIM00:<NA>** | Binary | 0.038 |
| **APTALK00** | Continuous | 0.090 |
| **APTALK00:<NA>** | Binary | 0.009 |
| **parentalMH** | Continuous | 0.206 |
| **parentalMH:<NA>** | Binary | 0.028 |
